# Intra and Inter-Rater Variability in the Interpretation of White Blood Cell Scintigraphy of Hip and Knee Prostheses

**DOI:** 10.3390/diagnostics14182043

**Published:** 2024-09-14

**Authors:** Giuseppe Campagna, Chiara Lauri, Ringo Manta, Roberta Ottaviani, Walter Davide Vella, Alberto Signore

**Affiliations:** 1 Department of Medical-Surgical Sciences and of Translational Medicine, Sant’Andrea Hospital, “Sapienza” University of Rome, 00161 Rome, Italy; chiara.lauri@uniroma1.it (C.L.); roberta_ottaviani@outlook.it (R.O.); alberto.signore@uniroma1.it (A.S.); 2 Department of Nuclear Medicine, Université Libre de Bruxelles, 1050 Brussels, Belgium; ringo.manta@ulb.be; 3 Nuclear Medicine Unit, Sant’Andrea Hospital, Via di Grottarossa 1035, 00189 Rome, Italy; wdvella@ospedalesantandrea.it

**Keywords:** white blood cell scintigraphy, periprosthetic infection, reproducibility, interpretation agreement, Gwet’s coefficient

## Abstract

**Background:** White blood cell (WBC) scintigraphy plays a major role in the diagnostic approach to periprosthetic infections. Although the procedure has been standardized by the publication of several guidelines, the interpretation of this technique may be susceptible to intra and inter-variability. We aimed to assess the reproducibility of interpretation between nuclear medicine physicians and by the same physician and to demonstrate that Cohen’s coefficient is more unstable than Gwet’s coefficient, as the latter is influenced by the prevalence rates. **Methods:** We enrolled 59 patients who performed a Technetium-99m WBC (^99m^Tc-WBC) scintigraphy for suspected hip or knee prosthesis infection. Three physicians, blinded to all patient clinical data, performed two image readings. Each WBC study was assessed both visually and semi-quantitatively according to the guidelines of the European Association of Nuclear Medicine (EANM). For semi-quantitative analysis, readers drew an irregular Region of Interest (ROI) over the suspected infectious lesion and copied it to the normal contralateral bone. The mean counts per ROI were used to calculate lesion-to-reference tissue ($\frac{L}{R}$) ratios for both late and delayed images. An increase in $\frac{L}{R}$ over time ($\frac{L}{R_{\mathrm{late}}}$> $\frac{L}{R_{\mathrm{delayed}}}$) of more than 20% was considered indicative of infection. Agreement between readers and between readings was assessed by the first-order agreement coefficient (Gwet’s AC_1_). Reading time for each scan was compared between the three readers in both the first and the second reading, using the Generalized Linear Mixed Model. **Results:** An excellent agreement was found among all three readers: 0.90 for the first reading and 0.94 for the second reading. Both inter- and intra-variability showed values ≥0.86. Gwet’s method demonstrated greater robustness than the Cohen coefficient when assessing the intra and inter-rater variability, since it is not influenced by the prevalence rate. **Conclusions:** These studies can contribute to improving the reliability of nuclear medicine imaging techniques and to evaluating the effectiveness of trainee preparation.

## 1. Introduction

Periprosthetic joint infection (PJI) is an infrequent but serious complication of prosthetic joint implantation. The infection commonly arises from the bacterial contamination of the prosthesis or the periprosthetic tissue during surgery or the colonization of the wound in the early postoperative period. The majority of PJIs occur within the first two years after surgery. Contiguous spread from a trauma or surgery at an adjacent location and hematogenous spread can also occur, although more rarely [1].

PJIs are responsible for 15% of total hip arthroplasty failures and 25% of revision total knee arthroplasty failures [2]. The incidence of PJI ranges from 0.7% to 2.4% for primary interventions and up to 20% for revision procedures [3,4]. In 2018, the incidence of hip and knee prosthesis infection in Italy was 7.8% and 18.9%, respectively [5]. With the increasing prevalence of joint prosthesis implantation procedures, the incidence of PJI is expected to rise over time [4,6]. For this reason, appropriate prophylactic measures for the prevention and the eradication of infections are nowadays mandatory in pre-operative phases, during arthroplasty as well as hospitalization, to reduce the risk of PJI and, consequently, the socio-economic burden [7,8,9,10].

Indeed, PJI can lead to repeated surgical interventions, prolonged hospitalization, chronic pain, functional disability, and significant economic burden, especially if the initiation of the treatment is delayed. Prompt and accurate detection of PJI is, therefore, crucial to prevent recurrence and to preserve mechanical joint function. Nevertheless, the diagnosis of PJI may be extremely challenging.

Clinical signs of infections may be absent or vague in a wide percentage of patients, serum inflammatory markers (i.e., erythrocyte sedimentation rate (ESR) or C-reactive protein (CRP)) demonstrated a quite low specificity and accuracy in the diagnosis of PJI, therefore, the diagnosis relies on a combination of clinical, biological and microbiological findings, the latter comprising periprosthetic cultures obtained on preoperative synovial fluid and/or intraoperative tissues [11,12,13,14]. Several multi-society joint consensus documents have been published in the past years, providing evidence-based recommendations and diagnostic flowcharts for PJI [11,12,13,14,15].

Imaging modalities are part of the arsenal of tools for the diagnosis and management of PJI; however, they are not included in all the definitions of PJI established by different societies [11,13,14]. Plain radiographs are usually performed as the initial imaging technique for patients with painful prosthetic joints to exclude prosthetic loosening or fracture but lack sensitivity and specificity for the diagnosis of PJI. Computed tomography (CT) and magnetic resonance imaging (MRI) may be used to guide further investigations but are not suited to reliably rule out infection [15]. Bone scintigraphy, using technetium ^99m^Tc-labelled bisphosphonates, allows the detection of an increased bone remodeling around the prosthetic joint with a high sensitivity. However, this technique lacks specificity for the diagnosis of PJI due to the numerous conditions characterized by an increased bone turnover, including aseptic inflammatory processes, fractures, and prosthetic loosening.

Radiolabeled white blood cell (WBC) scintigraphy, using in vitro labeling with [^111^In]In-oxine or [^99m^Tc]Tc-hexamethylpropyleneamine (HMPAO), is the gold-standard nuclear medicine imaging modality for the detection of bacterial infections that stimulate a neutrophil-mediated response [16]. Labeling with [^99m^Tc]Tc-HMPAO is generally preferred over [^111^In]In-oxine due to its more advantageous radiation characteristics for imaging, lower cost, wider availability, and lower radiation exposure to the patient [17].

Once labeled and re-injected into the patients, autologous WBCs migrate by diapedesis into the infection site where they accumulate over time, thus representing a surrogate marker of activated granulocytes. Given its well-known high specificity and accuracy in discriminating infection from sterile inflammation, WBC scintigraphy is the nuclear medicine imaging modality of choice for the differential diagnosis between PJI and aseptic loosening. This technique is nowadays widely adopted in clinical practice in many centers, depending on local availability and experience.

By using serial time points acquisitions (10 min–1 h, 3–4 h, and 24 h), the diagnostic accuracy increases and the number of false-positive findings decreases, particularly when applying decay-corrected acquisitions and image display in absolute counts [18,19,20,21].

For the particular case of musculoskeletal infection, a WBC scan can be combined with bone marrow imaging, using albumin colloids labeled with ^99m^Tc, to differentiate the physiological uptake of bone marrow from infection [16].

Planar antero-posterior images are usually sufficient for differential diagnosis between infection and sterile inflammation for prosthesis loosening, assuming images are correctly acquired and displayed as suggested by the guidelines of the European Association of Nuclear Medicine (EANM) guidelines published in 2018 [21].

Several guidelines have been proposed to standardize labeling procedures, acquisition protocols, interpretation criteria, quality controls, and safety procedures in all centers for WBC scintigraphy [21,22]. In a multicenter study by Erba et al. (2014) [23], WBC scintigraphy using these interpretation criteria yielded high sensitivity and specificity (93% and 100%) for the diagnosis of hip and knee prosthesis infection. If the visual analysis is doubtful, a semi-quantitative analysis can be performed. For the semi-quantitative analysis, regions of interest (ROIs) are drawn over the area of interest (usually with the highest uptake) and copied to a presumed normal reference tissue for both delayed and late phases. The mean counts per pixel in these ROIs are used to calculate the lesion-to-reference ($\frac{L}{R}$) ratio for the delayed and late phases. The examination is indicative of infection if the $\frac{L}{R}$ ratio increases by at least 20% over time, negative for infection if the $\frac{L}{R}$ ratio significantly decreases over time, and equivocal if the $\frac{L}{R}$ remains stable or slightly decreases. The infection is usually confirmed if the increase in $\frac{L}{R}$ between the delayed and late image is superior to 20%; however, this threshold is not standardized [24,25].

The purpose of this study was to assess the reproducibility of the interpretation of WBC scintigraphy on planar images, in suspected hip or knee prosthesis infection, both between different readers (inter-variability) and among the same reader (intra-variability), by demonstrating that Cohen’s coefficient is more unstable than Gwet’s coefficient.

## 2. Materials and Methods

### 2.1. Study Design and Patients

This was a blinded single-arm, single-center, retrospective study using three independent nuclear medicine readers. Fifty-nine WBC scintigraphies (GiPharma, Saluggia, Italy) performed for the suspicion of hip or knee prosthesis infection in our Nuclear Medicine Department between 15 September 2020 and 21 December 2021, were retrieved from our internal database, as stated in the paragraphs on statistical analysis and sample size.

All included patients had a microbiological or clinical diagnosis based on surgery or follow-up. This information was blinded to the three readers, since neither the calculation of diagnostic performance of WBC scintigraphy according to final diagnosis nor the comparison of the ability of the three readers were specific aims of this study.

All patients provided their consent by signing a specific “informed consent” form in which the clinical practice of scintigraphy was described. There were no minors in the study.

### 2.2. White Blood Cells Radiolabelling and Imaging

Autologous radiolabeled WBCs were prepared using Leukokit^®®^ (GiPharma, Saluggia, Italy) according to the EANM and Italian Association of Nuclear Medicine (AIMN) guidelines for labeling WBC with [^99m^Tc]Tc-HMPAO [21,22].

Whole-body and antero-posterior planar images were obtained 30 min (early images), then 3 h (delayed images) and 20 h (late images) post intravenous injection (p.i.) of 550–740 MBq of [^99m^Tc]Tc-HMPAO-WBC. Additional latero-lateral images were obtained in patients with suspicion of knee prosthesis infection. The acquisition time corrected for ^99m^Tc decay for early, delayed, and late images were, respectively, 100, 141, and 1007 s.

Images were acquired with a dual-head gamma camera (Forte, Philips, Milpitas, CA, USA) equipped with low-energy high-resolution collimators with a 20% energy window centered at the 140 keV (^99m^Tc) photon peak using a 512 × 512 matrix.

### 2.3. Readers

All WBC scintigraphies were assessed independently by three readers: A.S., C.L., and R.M. A.S. is a nuclear medicine physician with more than 30 years of clinical experience. C.L. is also a nuclear medicine physician with more than 10 years of clinical experience, while R.M. is a nuclear medicine trainee at the end of his training. A.S. and C.L. did not receive any specific training for the present study because their professional background was considered routine practice in the hospital, whereas training was planned for R.M. as part of his specialization in nuclear medicine.

WBC scintigraphies were enumerated from 1 to 59 and randomly assigned to the three readers for evaluating the inter-observer variability. The randomization of the studies was generated through a routine written in Statistical Analysis System (SAS) v. 9.4 by the department statistician (G.C.).

For determining the intra-observer variability, the readers had to assess the 59 scintigraphies twice, with a minimum 48 h (hours) interval between the first and second reading.

Readers were blinded to all patient data (including suspected infection site and microbiological results) and to the assessment made by the other readers. Each reader used, in autonomy, a dedicated room equipped with a Hermes workstation for image visualization.

The time needed by each reader to reach a conclusion of each WBC scan was also calculated using a stopwatch and recorded.

### 2.4. Image Interpretation

Planar images were displayed, in the total number of counts using the same intensity color scale both for delayed and late images, thereby avoiding operator bias in changing the intensity scale, according to the EANM guidelines [21]. Images were assessed visually and, in case of doubt, semi-quantitatively.

For the visual analysis, WBC scintigraphies were classified as binary data: “negative” for PJI when no uptake was observed or when the activity of the suspected focus was stable or clearly decreased from the delayed to the late images; “positive” when the activity of the suspected focus clearly increased from the delayed to the late images.

For semi-quantitative analysis, irregular ROIs were manually drawn over the suspected infectious lesion and copied to a presumed normal contralateral site as reference tissue.

The mean counts per pixel in these ROIs were recorded and used to calculate $\frac{L}{R_{\mathrm{late}}}$ and $\frac{L}{R_{\mathrm{delayed}}}$ ratios for both late and delayed images. The percentage variation of $\frac{L}{R}$ was calculated as:

$$\frac{\left(\frac{L}{R_{\mathrm{late}}}-\frac{L}{R_{\mathrm{delayed}}}\right)}{{\frac{L}{R}}_{\mathrm{delayed}}}$$

and was recorded as continuous data.

An increase in $\frac{L}{R}$ of more than 20% over time ($\frac{L}{R_{\mathrm{late}}}$ > $\frac{L}{R_{\mathrm{delayed}}}$) was considered indicative of infection. When $\frac{L}{R}$ ratios were decreased over time, were stable over time, or increased less than 20%, the patient was considered negative [24,25].

**Figure 1:**
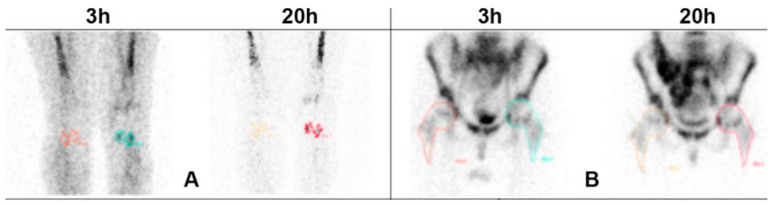
Anterior views acquired 3 h and 20 h p.i. of radiolabeled WBC. Images show an example of the methodology used for drawing regions of interest in knees (**A**) and hips (**B**). The colored areas, regardless of the colors, indicate the collecting area and therefore indicate the presence of inflammation.

An example of the methodology used for the semi-quantitative analysis is shown in Figure 1.

### 2.5. Statistical Analysis

The sample size was obtained from a table published by Sim et al. (2005) [26]. Data in the table were based on a Goodness-of-Fit formula provided by Donner et al. (1992) [27]. Considering a null value of kappa (k_0_ = 0.50), a power (1 − β) = 0.9, and a *p* < 0.05, the sample size required was n = 59.

Continuous variables are expressed as mean ± standard deviation (SD) and categorical variables are expressed as absolute frequency and percentage.

Gwet’s AC_1_ (first-order agreement coefficient), with 95%CI (95% Confidence Interval) was used to evaluate agreement among three readers (A.S. vs. C.L. vs. R.M.) when data were on a nominal scale (negative and positive) [28].

Gwet’s AC_1_ agreement coefficient was evaluated by *%magree* macro-SAS.

The agreement between the readers (A.S. vs. C.L., A.S. vs. R.M., and C.L. vs. R.M.) and the agreement between the first and second reading was assessed using both Cohen’s kappa [29] and Gwet’s AC_1_ in order to demonstrate the weakness of the Cohen’s kappa in presence of different prevalence rates. A value ≥ 0.80 indicates an excellent agreement between both Cohen’s kappa and Gwet’s AC_1_ coefficient.

$${AC}_{1}=\frac{p_{a-}p_{e\gamma }}{1-p_{e\gamma }}$$

where:

$p_{a}$: overall agreement probability

$p_{e\gamma }$: chance-agreement probability

Their computation formulas are as follows:

$$p_{a}=\frac{1}{n}\;\sum_{i=1}^{n}\left\{\sum_{q=1}^{Q}\frac{r_{iq}(r_{iq}-1)}{r(r-1)}\right\}$$

$$p_{e\gamma }=\frac{1}{Q-1}\;\sum_{q=1}^{Q}\pi_{q}(1-\pi_{q})$$

$$\pi_{q}=\frac{1}{n}\;\sum_{i=1}^{n}\frac{r_{iq}}{r}$$

where:

i: number of studies rated

q: number of categories in the rating scale

$r_{iq}$: number of raters who classified the ith studies into the qth category

r: total number of raters

$\pi_{q}$: probability that a rater classifies a study into categories q.

According to “Cohen’s paradox”, Cohen’s kappa coefficient is largely influenced by the prevalence rates, while Gwet’s AC_1_ coefficient is not affected and, therefore, more reliable, in the presence of a discrepancy between the percentages of positives and negatives [30,31].

The trait prevalence was calculated as the number of positive cases, as judged by the readers, in percentage of the total number of cases.

Reading time and percentage variation were both compared between readers and between first and second reading using the Generalized Linear Mixed Model (GLIMMIX) with repeated measurements and gamma distribution. *Post hoc* analysis was tested by the Tukey method.

The ranges of the degree of agreement for binary data are shown in Supplementary Table S1 [32,33,34,35].

Statistical analysis was performed using SAS version 9.4 TS Level 1 M8 and JMP PRO version 17 (SAS Institute, Cary, NC, USA).

A *p* value < 0.05 was considered statistically detectable.

## 3. Results

### 3.1. Patients

We included 59 adult patients (≥18 years old) who were referred to our Nuclear Medicine Department between September 2020 and December 2021 for the suspicion of hip (n = 32) or knee (n = 27) prosthesis infection, and underwent [^99m^Tc]Tc-HMPAO-WBC scintigraphy in our unit. The patient population included 30 females (mean age: 70.90 ± 11.07, years) and 29 males (mean age: 68.57 ± 12.21, years). The patient characteristics are shown in Table 1.

### 3.2. Qualitative Data

During the first reading, A.S. identified 5 positive and 54 negative cases, C.L. identified 4 positive and 55 negative cases, and R.M. identified 4 positive and 55 negative cases.

In the second reading, A.S. identified 4 positive and 55 negative cases, C.L. identified 4 positive and 55 negative cases, and R.M. identified 4 positive and 55 negative cases.

Gwet’s AC_1_ coefficients between all three readers were 0.90 (95%CI: 0.82 to 0.97) for the first reading and 0.94 (95%CI: 0.88 to 1.00) for the second reading, indicating excellent agreement.

Inter-variability between readers and intra-variability between the first and the second reading are shown in Table 2 and Table 3, respectively. Gwet’s AC_1_ coefficients were all ≥ 0.86, indicating an excellent agreement. Cohen’s Kappa coefficients were lower than Gwet’s AC_1_ for all analyses.

### 3.3. Reading Time for Qualitative Data

The comparisons of the reading time for the visual analysis between readers and between the first and second readings are shown in Table 4. A difference in reading time between the first and the second reading was statistically detectable for A.S. only (*p* = 0.009). All readers had a different time of reading during the first reading. The difference in time of reading was not statistically detectable only between A.S. and C.L. (*p* = 0.99).

### 3.4. Semi-Quantitative Analysis

Similarly, for the qualitative analysis, the results of the semi-quantitative analysis showed that at the first reading, A.S. identified 5 positive and 54 negative cases, C.L. identified 5 positive and 54 negative cases, and R.M. identified 4 positive and 55 negative cases.

In the second reading, A.S. identified 4 positive and 55 negative cases, C.L. identified 4 positive and 55 negative cases, and R.M. identified 5 positive and 54 negative cases.

The statistical analysis of these results is the same as for the qualitative analysis and is shown in Table 2 and Table 3.

## 4. Discussion

Our study shows an excellent intra and inter-observer agreement among the three readers. They showed almost identical results to each other, indicating successful training in WBC scintigraphy interpretation.

Beyond the excellent reproducibility achieved among the three physicians from our department, we used the WBC scintigraphy as a model for the assessment of the inter- and intra-observer reproducibility that could be generalized to other nuclear medicine techniques. Over the past decades, the role and indications of nuclear medicine techniques have become more clearly defined in the diagnostic approaches to various diseases. As reported in a large number of publications, these techniques continue to demonstrate higher diagnostic performance. Despite these great improvements, inter-observer (and intra-observer) reproducibility in reporting remains essential to consider an imaging technique useful in daily practice. Therefore, inter and intra-observer studies play an important role in helping practitioners improve their results and allow us to compare the assessment between physicians from different countries, ultimately contributing to improving the consistency and reliability of nuclear medicine techniques. Additionally, this approach could be used to evaluate if the preparation of a trainee was effective.

Tondeur et al. (2008) [36] and Fernandez et al. (2008) [37] evaluated the inter-observer reproducibility of the interpretation of WBC scintigraphy. In the study of Tondeur et al., twenty physicians from various centers in Belgium assessed 10 scans for suspected infections (5 osteoarticular infections, 2 vascular graft infections, 1 intra-abdominal, and 2 fevers of unknown origin), revealing a poor inter-observer reproducibility. In the study by Fernandez et al., physicians assessed 70 scans for suspicion of osteomyelitis. The inter-observer reproducibility was moderate (63% prevalence of agreement, *κ* = 0.50) at 4 h and good (80% prevalence of agreement, *κ* = 0.74) at 24 h. Interestingly, in both studies, they used an unvalidated and less precise scoring system, categorizing results as “low probability”, “intermediate probability”, “high probability” and so forth, for infection. In contrast, readers in our study used the recently published interpretation criteria for the diagnosis of infection (including PJI) with WBC scintigraphy [21,22,23]. These criteria provide a standardized method to estimate visually and quantitatively the WBC accumulation over time. Similar criteria were used in a multicenter study, with the primary objective of comparing anti-granulocyte imaging with [^99m^Tc]Tc-HMPAO WBC scintigraphy in patients with peripheral osteomyelitis. The authors reported a high reproducibility for both techniques among the three blinded readers [38]. These different results may also be explained by the different levels of expertise of the participating physicians, the different acquisition protocols, or the different statistical methodologies. Nevertheless, establishing well-standardized interpretation criteria may lead to a higher degree of agreement between observers and a minimized variation in reporting. In nuclear medicine, there are still relatively few techniques with well-standardized interpretation criteria.

Reading time for the qualitative analysis was recorded using a stopwatch to determine whether any disparities in results between two readers could be attributed to reading times. Statistically detectable discrepancies emerged during the first reading between the pairs of readers, while in the second reading, the differences can only be seen between the more experienced physicians (A.S. and C.L.) and the nuclear medicine trainee (R.M.). This, in our local experience, would suggest that expert readers take more time than less experienced physicians to assess the images and provide a definite conclusion.

The semi-quantitative analysis is strictly dependent on the ROI’s size and shape. In this study, ROIs were manually drawn around the presumed area of infection and copied around the presumed normal contralateral background. In this experience, we demonstrate that ROIs were similarly drawn by all readers. Semi-quantitative analysis confirms excellent agreement between readers both when considering pairwise comparisons and when comparing first and second readings. Similarly, no differences were found between the qualitative and semi-quantitative analysis, highlighting the importance of qualitative interpretation of images when correctly acquired with time corrected for isotope decay. Indeed, semi-quantitative analysis should be restricted to those few doubtful cases, as described by Lauri et al. (2020) [24].

It is important to note that the primary endpoint was to assess the agreement between the physicians in terms of inter- and intra-observer reproducibility and not to assess the diagnostic performance of each of the three readers. The three physicians did not have access to the patients’ clinical data, i.e., the site of suspected infection, previous imaging, laboratory results, and microbiological/histopathological results.

As a possible limit, the use of a stopwatch to record reading time may have created a time constraint that could have influenced the physicians’ interpretation and final diagnosis. In addition to these aspects, in our study, we used two distinct tests, Gwet’s AC_1_ and Cohen’s Kappa, to emphasize that Gwet’s method demonstrates greater robustness when assessing intra and inter-rater variability, since it is not influenced by the prevalence rate, namely in the presence of a discrepancy between the percentages of positives and negatives [39,40].

## 5. Conclusions

We used WBC scintigraphy as a model for the assessment of the intra and inter-observer reproducibility of qualitative and semi-quantitative interpretation criteria. Our study shows a high intra and inter-observer reproducibility for the interpretation of WBC scintigraphy in patients with hip and knee prosthesis infection using the current standardized interpretation criteria. This study confirms the validity of image acquisition and interpretation criteria established by EANM.

Investigation of inter- and intra-observer reproducibility could be translated to other imaging techniques, contributing to improving their reliability and evaluating the effectiveness of trainee preparation.

Gwet’s method demonstrated greater robustness than the Cohen coefficient when assessing intra and inter-rater variability.

## Figures and Tables

**Table 1:** Demographic characteristics and clinical parameters of n = 59 patients.

Parameter	Mean ± SD
Sex *	
Female	30 (50.85)
Male	29 (49.15)
Age (years)	69.75 ± 11.61

* data presented as n (%).

**Table 2:** Agreement between readers (inter-rater variability) for qualitative data.

	A.S. vs. C.L.	A.S. vs. R.M.	C.L. vs. R.M.
First reading			
Cohen’s kappa	0.64 (0.26 to 1.00)	0.16 (−0.22 to 0.54)	0.20 (−0.22 to 0.61)
Gwet’s AC_1_	0.94 (0.87 to 1.00)	0.86 (0.75 to 0.97)	0.88 (0.79 to 0.98)
Second reading			
Cohen’s kappa	0.73 (0.38 to 1.00)	0.46 (0.02 to 0.91)	0.46 (0.02 to 0.91)
Gwet’s AC_1_	0.96 (0.91 to 1.00)	0.92 (0.84 to 1.00)	0.92 (0.84 to 1.00)

**Table 3:** Agreement between the first and the second reading (intra-rater variability) for qualitative data.

Reader	A.S.	C.L.	R.M.
Cohen’s Kappa (95%CI)	0.88 (0.65 to 1.00)	0.73 (0.38 to 1.00)	0.20 (−0.22 to 0.61)
Gwet’s AC_1_ (95%CI)	0.98 (0.94 to 1.00)	0.96 (0.91 to 1.00)	0.88 (0.79 to 0.98)

**Table 4:** Comparisons of the reading time between readers and between first and second reading for qualitative data.

Reader	A.S.	C.L.	R.M.	A.S. vs. C.L. *p*	A.S. vs. R.M. *p*	C.L. vs. R.M. *p*
First reading time				0.04	<0.0001	0.009
Mean ± SD	19.54 ± 17.09	13.71 ± 7.16	9.05 ± 4.15			
(95%CI)	(15.09 to 24.00)	(11.85 to 15.58)	(7.97 to 10.13)			
Second reading time				0.99	0.0003	<0.0001
Mean ± SD	13.69 ± 15.27	13.19 ± 8.84	7.88 ± 3.59			
(95%CI)	(9.72 to 17.67)	(10.88 to 15.49)	(6.95 to 8.82)			
*p*	**0.009**	0.99	0.93			

Time is expressed in seconds.

## Data Availability

The original data presented in the study are openly available in the named database “DB_intra and inter.xslx”.

## Supplementary Materials

The following supporting information can be downloaded at: https://www.mdpi.com/article/10.3390/diagnostics14182043/s1, Table S1: Ranges of agreement measures, according to some authors.
