# Supplementary material for: Intra and Inter-Rater Variability in the Interpretation of White Blood Cell Scintigraphy of Hip and Knee Prostheses
Source: Diagnostics (Basel). 2024 Sep 14;14(18):2043. doi: 10.3390/diagnostics14182043 (PMC11431631; doi:10.3390/diagnostics14182043)
Supplement: Supplementary file 1 [file diagnostics-14-02043-s001.zip › diagnostics-3167439-supplementary.pdf]

Supplementary Table S1 - Ranges of agreement measures, according to some authors

Fleiss J.L.	Landis J.R. and Koch G.G.	Altman D.G.	Cicchetti D.V.
	<0.00: Poor		
<0.40: Poor	0.00 to 0.20: Slight	<0.20: Poor	
	0.21 to 0.40: Fair	0.21 to 0.40: Fair	<0.40: Poor
0.40 to 0.75: Intermediate to Good	0.41 to 0.60: Moderate	0.41 to 0.60: Moderate	0.40 to 0.59: Fair
	0.61 to 0.80: Substantial	0.61 to 0.80: Good	0.60 to 0.74: Good
≥0.75: Excellent	0.81 to 1.00: Almost Perfect	0.81 to 1.00: Very Good	0.75 to 1.00: Excellent
